# Assessment of Bone Metastases in Patients with Prostate Cancer—A Comparison between ^99m^Tc-Bone-Scintigraphy and [^68^Ga]Ga-PSMA PET/CT

**DOI:** 10.3390/ph10030068

**Published:** 2017-07-31

**Authors:** Lena Thomas, Caroline Balmus, Hojjat Ahmadzadehfar, Markus Essler, Holger Strunk, Ralph A. Bundschuh

**Affiliations:** 1 Klinik und Poliklinik für Nuklearmedizin, Universitätsklinikum Bonn, D-53127 Bonn, Germany; caroline.balmus@gmx.de (C.B.); hojjat.ahmadzadehfar@ukbonn.de (H.A.); markus.essler@ukbonn.de (M.E.); ralph.bundschuh@ukb.uni-bonn.de (R.A.B.); 2 Radiologische Klinik, Universitätsklinikum Bonn, D-53012 Bonn, Germany; holger.strunk@ukbonn.de

**Keywords:** prostate cancer, bone scan index, bone scintigraphy, PSMA-PET/CT

## Abstract

Purpose: Bone scintigraphy is the standard of reference in bone metastases in prostate cancer patients. However, new radiotracers employed in prostate-specific membrane antigen (PSMA)-ligands has led to the growing importance of PET/CT as diagnostic tool. The aim of our study was to investigate the difference between bone scan and PSMA-PET/CT for the detection of bone metastases in prostate cancer. Methods: Thirty patients with bone metastases originating from prostate cancer were examined by ^99m^Tc-MDP bone scan and ^68^Ga-PSMA-PET/CT within an average of 21 days. Bone scans were analyzed visually according to the number of lesions and using the software package ExiniBONE by Exini Diagnostics. PET/CT data was analyzed visually. Numbers of detected lesions were compared for the different methods for the whole patient and for different regions. In addition, results were compared to serum prostate-specific antigen (PSA), alkaline phosphatase (ALP), bone alkaline phosphatase (bALP), pro gastrin releasing peptide (pGRP) and eastern cooperative oncology group (ECOG) performance status. Results: In the bone scans, visual and semiautomatic lesion detection showed similar results with an average of 19.4 and 17.8 detected bone lesion per patient. However, in PSMA-PET/CT, on average double the numbers of lesions (40.0) were detected. The largest differences were found in the thorax and pelvis, which can be explained by the advantages of tomographic imaging. Bland-Altman analysis showed greater differences in patients with large numbers of bone metastases. Conclusion: No significant difference was found when using semiautomatic analysis compared to visual reading for bone scans. Fewer bone metastases were detected in bone scans than in PSMA-PET/CT. However, in none of our patients would the difference have led to clinical consequences. Therefore, it seems that for patients undergoing PSMA-PET/CT, there is no need to perform additional bone scans if the appropriate PET/CT protocols are applied.

## 1. Introduction

With 14% of men being diagnosed with prostate cancer at some point during their life, this type of cancer represents one of the most common malignancies in the United States and Europe [1]. While 98% have a general five-year survival rate, this decreases quickly with the diagnosis of distant metastases [1]. The most common site for distant metastases is the skeletal system, and nearly 85% of patients with fatal prostate cancer show bone metastases [2]. In clinical workflow, a bone scan is the most common and cost effective modality for the diagnosis of bone metastases [3]. For quantitative assessment, e.g., in tumor response evaluation, the bone scan index (BSI) has been developed [4]. While the BSI was initially calculated manually, a semiautomatic algorithm was developed by EXINI Diagnostics [5]. This algorithm, based on the neuronal network technique, detects suspected metastatic lesions and calculates the BSI based on its findings. The BSI was consequently found to be associated with overall survival in newly diagnosed prostate cancer patients [6]. In addition, the automated BSI was found to be reproducible for standardizing quantitative changes in the bone scans of prostate cancer patients in an recent analysis study [7], and therefore seems to be of growing interest for such applications [8].

While bone scans can solely detect bone metastases, positron emission tomography (PET) has become important in the diagnosis of patients with prostate cancer [9]. Most radiotracers in use for PET have the advantage of detecting any location of active tumor tissue compared to the bone scan. Therefore, this method may be preferable in the case of suspicion of any metastatic site, e.g., in the case of increased serum prostate-specific antigen (PSA). The most common oncological PET-tracer [^18^F]fluorodesoxyglucose (FDG) was found to be able to influence the clinical management of patients with prostate cancer, although its influence was found to be lower in prostate cancer compared to other cancer types [10]. The limited use of this diagnostic method is due to its lack of specificity with uptake in normal tissue (e.g., bladder), benign prostate hyperplasie inflammation, and infection. Also, FDG-PET may give a false negative in the case of inactive bone metastases [11]. In light of this, a combination of FDG and [^18^F]fluoride was used as cocktail to increase the sensitivity for bone metastases [12]. Minamimoto and colleagues recently compared combined FDG- and [^18^F]fluoride-PET/CT to bone scans and whole-body MRI in 30 patients with prostate or breast cancer [13]. They found that PET/CT was superior to bone scan and whole-body MRI for the evaluation of the extent of bone metastases in these patients.

^68^Ga-labeled ligands to prostate-specific membrane antigen (PSMA) were introduced for the diagnosis of prostate cancer patients in recent years [14]. Consequently, [^68^Ga]Ga-PSMA-PET was found to have high sensitivity and specificity in the diagnosis of primary [15] or recurrent prostate cancer [16]. PSMA-PET, as a hybrid modality with computed tomography (PET/CT) and magnetic resonance tomography (PET/MR), has been evaluated for the detection of bone metastases [17]. Recently, original studies have been published comparing bone scans and [^68^Ga]Ga-PSMA-PET for the detection of bone metastases [18,19]. However, in these works, only visual interpretation was employed in the analysis of bone scans. Another study was also published recently by Bieth et al., in which the authors propose new parameters for the quantification of the osseous tumour burden in prostate cancer based on PSMA-PET/CT data [20]. In this study, again a main advantage of the semiautomatic parameter was shown to be the low interobserver variability and the short time which is necessary for assessment.

Therefore, in the presented study we also compared visual interpretation of bone scan data with PSMA-PET/CT data with respect to the detectability of bone lesions, while we also included a semiautomatic tool for lesion detection developed by EXINI Diagnostics AB (Lund, Sweden).

## 2. Results

### 2.1. Comparison between Bone Scan and PET/CT

**Figure 1:**
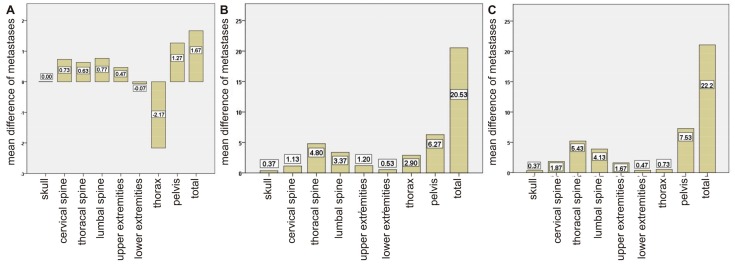
Graph of the average difference of detected lesions based on regions for (**A**) visual analysis of bone scans and semiautomatic analysis (EXINI) of bone scans, (**B**) visual analysis of bone scans and [^68^Ga]Ga-PSMA-PET/CT, (**C**) semiautomatic analysis of bone scans (EXINI) and [^68^Ga]Ga-PSMA-PET/CT.

**Figure 2:**
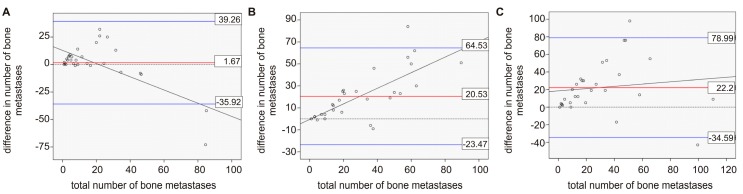
Bland-Altman plot including the linear trend of the comparison of (**A**) visual analysis of bone scans and semiautomatic analysis (EXINI) of bone scans, (**B**) visual analysis of bone scans and [^68^Ga]Ga-PSMA-PET/CT, (**C**) semiautomatic analysis of bone scans (EXINI) and [^68^Ga]Ga-PSMA-PET/CT.

In 30 patients, bone scans with visual analysis and semiautomatic lesions detection was compared to each other and to the results of a visual analysis of [^68^Ga]Ga-PSMA-PET/CT. On average, 19.4 lesions were detected by visual analysis and 17.8 were detected by EXINI software (version 1.8.5, EXINI Diagnostics AB, Lund, Sweden) in bone scans, while in [^68^Ga]Ga-PSMA-PET/CT on average 40.0 bone lesions were detected. The individual numbers of lesions for each patient can be found in Supplementary Table S1. When comparing the visual analysis of bone scans with the semiautomatic lesion detection of bone scan images, in most regions the number of detected lesions by visual analysis was larger than that detected by EXINI software. Only in the thorax and the lower extremities was this result found to be the opposite. The greatest overall differences were found in the thorax and pelvis. A detailed overview of the different regions can be found in Figure 1A. In addition, we found that differences between the two methods became larger with increasing number of metastases in the patient. In the Bland-Altman plot, only the two patients with the highest number of lesions (>80 metastases in the bone scan) were outside the limits of agreement (Figure 2A).

A comparison of the visual analysis of the bone scan with the visual analysis of the [^68^Ga]Ga-PSMA-PET/CT showed that, with 40.0 lesions on average, the PET/CT examination detected about double the number of lesions that were detected by the bone scans. In 27 of the 30 cases (90%), the number of detected lesions by PET/CT was equal to or larger than number of lesions detected in the bone scans. The individual numbers of lesions for each patient can be found in Supplementary Table S1. The lowest differences between the two modalities were found in the skull and the extremities; an overview of the differences across all regions can be found in Figure 1B. In the Bland-Altman plot, all but two patients are within the borders of agreement (Figure 2B). Again, the difference between the two methods is shown to increase with higher number of overall lesions. Comparing [^68^Ga]Ga-PSMA-PET/CT to semiautomatic lesion detection by EXINI leads to similar results as the comparison of the visual analysis of the bone scan to PET/CT. The differences between the various regions also exhibit results similar to the comparison of PET/CT to the visual analysis of the bone scan (Figure 1C). In the Bland-Altman plot, there was just one patient outside the borders of agreement (Figure 2C). As can also be seen in the Bland-Altman plot, merely three (visual analysis) and two (semiautomatic analysis) of the patients undergoing PSMA-PET/CT showed less metastases than that detected by the bone scan. In no single case had the bone scan found metastases in a region in which PSMA PET/CT did not detect bone lesions.

### 2.2. Comparison of Imaging and Serum Biomarkers

Comparing results from the bone scan, we found a statistically significant correlation between the number of metastases found in visual interpretation and serum PSA (*r* = 0.550, *p* = 0.002), serum alkaline phosphatase (ALP) (*r* = 0.651, *p* < 0.001), and serum bone alkaline phosphatase (bALP) (*r* = 0.649, *p* < 0.001). Similar results were found for the correlation between semiautomatic findings in the bone scan and serum PSA (*r* = 0.507, *p* = 0.004), serum ALP (*r* = 0.805, *p* < 0.001) and serum bALP (*r* = 0.853, *p* < 0.001). For the number of bone lesions in PSMA-PET/CT, we found a similar correlation with serum PSA (*r* = 0.593, *p* = 0.001), but a lower correlation with serum ALP (*r* = 0.459, *p* = 0.012) and serum bALP (*r* = 0.398, *p* = 0.033). The serum levels for the individual patients are shown in Supplementary Table S1.

None of the imaging modalities showed a significant correlation with patient age, serum pro gastrin releasing peptide (pGRP), or the eastern cooperative oncology group (ECOG) performance status.

## 3. Discussion

**Figure 3:**
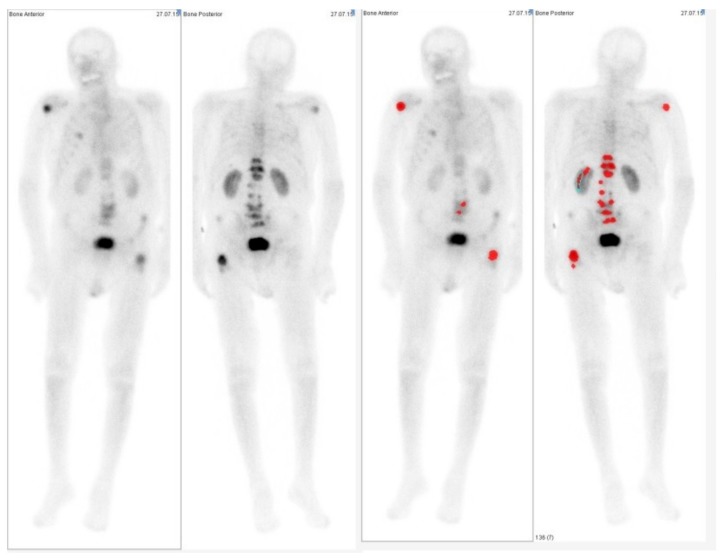
Example of a case in which the semiautomatic tool uptake in the renal system was classified as metastasis (red marked areas).

When comparing the semiautomatic algorithm (EXINI software) for the ability to detect lesions in patients with prostate carcinoma, we found only minor differences in most of the patients. Only in two cases presenting more than 80 detected lesions were the values outside the limits of agreement of the Bland-Altman analysis. Comparing different regions in the body, we found the greatest discrepancies to be in the thorax, where EXINI software detected on average two lesions more per patient as found by the clinical observer. However, careful control of the semiautomatic algorithm in this region is necessary, as in one case uptake in the kidney was detected as metastases by the software (Figure 3). The region with the second highest discrepancy was the pelvis, where complex bone anatomy and a highly active bladder make the analysis difficult for the human observer as well as for the software when only using planar images from the front and back. In clinical routine, often lateral images or tomographic images using single photon emission tomography (SPECT) technique are acquired. However, as the software package only takes into account planar whole-body images, the clinical observer in our study also only used these images; although, in the clinical examination other data was acquired as well. These limitations of the semiautomatic detection method are consistent with the results reported in other studies [7]. Anand et al. found that in a study with simulated data, that lesions superimposed on the bladder are not detected by the automated-BSI.

**Figure 4:**
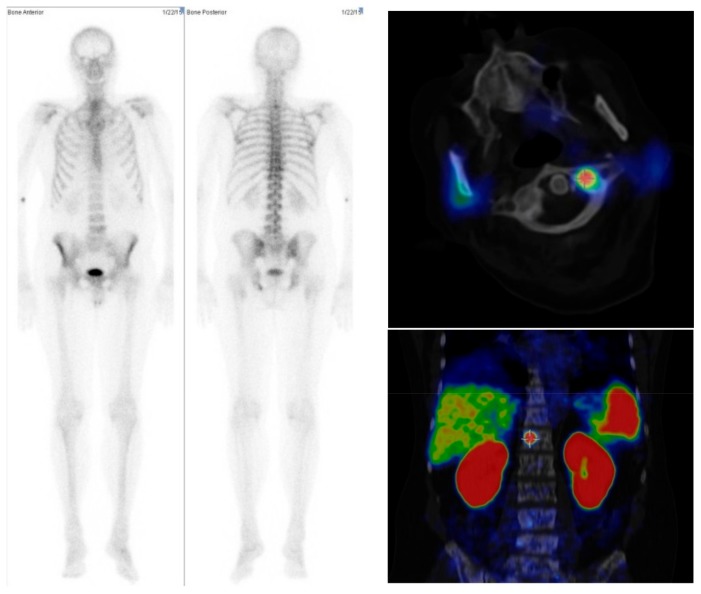
Example of a case in which the semiautomatic tool showed no lesions suspicious for metastases in the bone scan images (**Left** and **Middle**), while the visual analysis suspected bone lesions in the cervical, thoracic, and lumbar spine. PSMA-PET/CT agreed with these findings (**Right**).

Also mainly in patients with a low number of metastases, the agreement between the numbers of lesions detected by EXINI software and the human observer was good. We had five patients in which the semiautomatic algorithm did not detect any bone lesion, while visual analysis of the bone scan and [^68^Ga]Ga-PSMA-PET/CT was suspicious for metastases (Figure 4). Therefore, in clinical routine careful review of the results from semiautomatic methods is necessary. In this context, it also needs be mentioned that in the visual interpretation of bone scans, differences between different observers may also occur. In a Swedish multicentric study with 37 different observers, Sadik and colleagues found only a moderate interobserver agreement, with false-negative errors presenting a major problem [21]. Differences in the patients with a higher number of lesions are often caused by the fact that metastases could not be separated clearly from each other. However in such patients the precise number of lesions is not of clinical relevance. However, Ulmert et al. found a similar result, that the EXINI software tends to underestimate the bone scan index in patients with extremely extended bone metastases [6].

Like other recently published studies [18,19], we found that significantly more lesions were detected in the PET/CT examination compared to the bone scan. Compared to these two studies, we included not only the visual analysis of the bone scan, but also a semiautomatic algorithm which allows for faster analysis and reduces interobserver variability. The findings for both methods (visual and semi-quantitative analysis) are probably caused by the fact that a tomographic imaging modality was compared to a planar method. However, the important result is that no relevant information concerning bone lesions seems to be missed when using PSMA-PET/CT. When comparing this to the visual interpretation of the bone scan, and to the semiautomatic software package, in just three and two patients, respectively, was the number of detected bone lesions lower in PSMA-PET/CT than in the bone scan. In none of these patients did the bone scan show lesions in a region where the PET/CT results were negative. Therefore, the use of PSMA-PET/CT for the diagnosis of bone metastases would have not altered the clinical management of any patient if bone scans had not been performed. Also, PSMA-PET/CT is more convenient for the patient, as scans can be performed as early as 30 min after the injection of the radiopharmaca, while for the bone scans a waiting time of at least 120 min is recommended. PSMA-PET/CT showed very good ability in lymph node staging and the diagnosis of other extra-osseous lesions [16]. Therefore, a more extended diagnosis can be performed using one examination e.g., in the case of a biochemical relapse. However, costs are significantly higher for the PET/CT examination than for the bone scan. Thus, we do not suggest that bone scans should be replaced by PSMA-PET/CT in all cases, but that bone scans can be avoided if PSMA-PET/CT is performed primarily. In this context, it is important to perform the PET/CT as a whole-body examination, so as to not miss any metastases in the extremities, despite the fact that bone lesions in distant extremities are rare due to the low amount of red bone marrow. Even though performing the PET/CT scan as a whole-body examination may increase the scan time a little (depending on the protocol; 2–4 additional bed positions requiring 2–3 min each), it is still much more convenient for the patient. Furthermore, from an economic point of view, the whole-body PET/CT examination is still cheaper than performing two different examinations, including the cost for the second radiopharmaca and the additional 3–4 h of time cost.

A limitation of this study is that we had no gold standard for the detection of real metastases; therefore, it cannot be said that ^68^Ga-PSMA-PET/CT is more sensitive in the detection of lesions, as we cannot define a number of false-positive lesions. However, as the bone scan is considered as a kind of gold standard, we can say that PSMA-PET/CT does not miss clinically relevant information concerning bone lesions in our patients. 

Another limitation may be the heterogeneity in the patient cohort. However, we are interested in having a cohort which represents patients in the clinical routine. Therefore, the choice of our retrospective analysis to include consecutive patients with only few exclusion criteria seems to be justified.

## 4. Materials and Methods

### 4.1. Patient Population and Treatment

Thirty patients with biopsy-proven prostate carcinoma, that had been referred to our department for therapy of bone metastasis with radium-223-dichlorid (^223^RaCl_2_, Bayer Vital GmbH, Leverkusen, Germany), were retrospectively analyzed in this study. All patients underwent bone scans and [^68^Ga]Ga-PSMA PET/CT examination within an average interval of 21 days before planned therapy. In 22 patients, therapy with ^223^RaCl_2_ was initiated; in seven patients a therapy with [^177^Lu]Lu-PSMA-617 was initiated, as organ metastases were found in the PET/CT examination; and one patient was found not suitable for a therapy in our department. In all patients, serum blood levels for PSA were obtained at the time as the bone scan or PET/CT examination. In all patients except one serum levels of ALP and bALP were taken, and in 15 of the 30 patients serum level of pGRP was additionally obtained. At the same time point, ECOG performance status was evaluated for 15 of the 30 patients [22]. All procedures performed in retrospective analysis involving patient data were in accordance with the ethical standards of the institutional and/or national research committee and with the 1964 Helsinki declaration and its later amendments or comparable ethical standards. As a retrospective analysis, the ethics statement is waived in our institution by the institutional ethics committee. All patients gave written and informed consent for all imaging procedures and treatment.

### 4.2. Imaging

Bone scan was performed using on average 646.6 MBq technetium-99m-methylenediphosphonate (MDP, PoltechMDP, Polatom Otwock, Poland, order Nr. 900 200 16, reseller: ROTOP Pharmaka AG, Dresden, Germany). Subsequently, 120 to 240 min post-injection, planar whole-body images were acquired. Depending on the findings and the clinical questions, additional planar or SPECT images were acquired. Bone scans were performed on the following systems: Philips IRIX three-head-camera (Philips Medical Systems, Amsterdam, The Netherlands) and MEDISO Any Scan S (Mediso Medical Imaging Systems Ltd., Budapest, Hungary).

PET scan was performed using on average 141.2 MBq [^68^Ga]Ga-PSMA-11, ABX Radeberg, Germany, order Nr. 9920.0001). Data were acquired with a Biograph 2 PET/CT scanner (Siemens Medical Solutions, Erlangen, Germany) on average 77.1 min post-injection. First, a low-dose CT (16 mAs, 130 kV) from the base of the skull to mid thigh was acquired. The PET scan was acquired over the same area. CT data was reconstructed in 512 to 512 matrices with a thickness of 5 mm. PET data was reconstructed in 128 to 128 matrices with a thickness of 5 mm, using an attenuation-weighted ordered subsets expectation maximization algorithm performing attenuation and scatter correction as implemented by the manufacturer.

### 4.3. Data Analysis

Planar bone scan images were analyzed by an experienced reader. The number of metastases was estimated according to nine different areas: skull, cervical spine, thoracic spine, lumbar spine, upper extremities, lower extremities, thorax, and pelvis. For the semiautomatic analysis of the bone scans, EXINI bone^BSI^ software (Exini Diagnostics, AB, Lund, Sweden) was used. The technical details of the software have been described elsewhere [6]. For statistical comparison, the number of metastases in each of the nine different regions detected by the software was used.

PSMA-PET/CT was analyzed visually as well, and the numbers of detected lesions were estimated according to the abovementioned nine different body regions. A visual analysis of the bone scan images was performed on printed images and or in Interview Fusion software (Mediso Medical Imaging Systems Ltd., Budapest, Hungary); PET analysis was performed in Interview Fusion software as well.

### 4.4. Statistical Analysis

All statistical analyses were performed using IBM SPSS Statistics 23 (IBM, Armonk, NY, USA). For a quantitative comparison of the visual analysis of the bone scans, the semiautomatic analysis, and PSMA-PET/CT, Bland-Altman plots were calculated including the 95% limit of agreement [23]. Absolute and relative differences in numbers of metastases found for the whole patient and each region were calculated and compared as well. In addition, imaging findings were correlated with patient age, and blood serum levels of PSA, ALP, and bALP by Spearman correlation.

## 5. Conclusions

Our results suggest that the bone scan is not mandatory when [^68^Ga]Ga-PSMA-PET/CT is performed. In quantifying the number of lesions found via semiautomatic detection by EXINI software, this method was found to be adequate if the tumor load of the patient was not too extended. However, in detecting whether bone metastases were present at all, the semiautomatic algorithm failed in five patients. Therefore, in clinical routine a careful review of the results of the semiautomatic algorithm is necessary.

## Supplementary Materials

The following are available online at www.mdpi.com/1424-8247/10/3/68/s1. Table S1: Serum levels of the different biomarkers as well as the numbers of bone lesions (#bl) detected by visual analysis of the bone scans (bsv) and by the semiautomatic software package (bse), as well as in the PET/CT examination for individual patients.
