# Supplementary material for: Assessment of Bone Metastases in Patients with Prostate Cancer—A Comparison between 99mTc-Bone-Scintigraphy and [68Ga]Ga-PSMA PET/CT
Source: Pharmaceuticals (Basel). 2017 Jul 31;10(3):68. doi: 10.3390/ph10030068 (PMC5620612; doi:10.3390/ph10030068)
Supplement: Supplementary file 1 [file pharmaceuticals-10-00068-s001.docx]

**Supplementary Material:** **Assessment of Bone Metastases in Patients with Prostate Cancer—A Comparison between 99mTc-Bone-Scintigraphy and [68Ga]Ga-PSMA PET/CT**

Lena Thomas Caroline Balmus, Hojjat Ahmadzadehfar, Markus Essler, Holger Strunk and Ralph A. Bundschuh

**Table S1.** Serum levels of the different biomarkers as well as the numbers of bone lesions (#bl) detected by visual analysis of the bone scans (bsv) and by the semiautomatic software package (bse), as well as in the PET/CT examination for individual patients.

| **Patient No.** | **Patient Age** | **ALP [U/L]** | **PSA [ng/mL]** | **bALP [µg/mL]** | **pGRP [pg/mL]** | **ECOG** | **#bl**  **bsv** | **#bl**  **bse** | **#bl**  **PET** |
| --- | --- | --- | --- | --- | --- | --- | --- | --- | --- |
| 1 | 69 | 28 | 1.15 | 3.4 | 63.12 | 0 | 5 | 0 | 9 |
| 2 | 65 | 59 | 77 | 9.8 | 73.4 | 0 | 9 | 0 | 26 |
| 3 | 75 | 118 | 86.9 | 38.3 | 55.84 | 0 | 9 | 9 | 9 |
| 4 | 68 | 207 | 30.5 | 59.5 | 63.95 | 0 | 25 | 24 | 43 |
| 5 | 66 | 58 | 145 | 10.5 | 50.72 | 0 | 35 | 9 | 85 |
| 6 | 77 | 58 | 3.89 | 13.8 | / | / | 2 | 0 | 4 |
| 7 | 80 | 73 | 179 | 16.9 | 50.59 | 1 | 7 | 6 | 11 |
| 8 | 78 | 43 | 70.9 | 11.3 | 52.46 | 1 | 9 | 2 | 32 |
| 9 | 57 | 65 | 2.56 | 10.9 | / | / | 8 | 0 | 20 |
| 10 | 75 | 55 | 11.8 | 10.7 | 53.91 | 0 | 2 | 1 | 4 |
| 11 | 77 | / | 335 | / | / | / | 64 | 106 | 115 |
| 12 | 70 | 61 | 1.31 | 8.8 | 43.02 | 0 | 1 | 1 | 1 |
| 13 | 73 | 73 | 85.4 | 18.5 | 33.79 | 0 | 38 | 6 | 57 |
| 14 | 73 | 123 | 594 | 46.4 | / | / | 16 | 17 | 22 |
| 15 | 66 | 205 | 122 | 42.4 | 32.4 | 0 | 7 | 8 | 20 |
| 16 | 78 | 90 | 21.1 | 14.8 | 39.1 | 0 | 10 | 6 | 18 |
| 17 | 79 | 65 | 124 | 11.8 | / | / | 15 | 14 | 40 |
| 18 | 74 | 78 | 73.7 | 14.5 | / | / | 15 | 8 | 61 |
| 19 | 73 | 64 | 2.25 | 12.1 | / | / | 7 | 3 | 33 |
| 20 | 73 | 74 | 10.2 | 12.5 | / | / | 2 | 2 | 4 |
| 21 | 64 | 59 | 0.01 | 8.8 | 52.13 | 0 | 4 | 1 | 4 |
| 22 | 76 | 226 | 8.68 | 80.9 | 75.94 | 1 | 42 | 50 | 33 |
| 23 | 80 | 158 | 72.5 | 54 | 106.83 | 1 | 38 | 25 | 62 |
| 24 | 77 | 359 | 264 | 108.9 | / | / | 42 | 51 | 65 |
| 25 | 81 | 85 | 379 | 10.3 | / | / | 7 | 0 | 32 |
| 26 | 71 | 142 | 86 | 30.1 | / | / | 39 | 14 | 33 |
| 27 | 43 | 135 | 19.1 | 36.2 | / | / | 30 | 10 | 86 |
| 28 | 68 | 585 | 851 | 225 | / | / | 48 | 121 | 78 |
| 29 | 71 | 251 | 534 | 105.5 | / | / | 31 | 38 | 93 |
| 30 | 73 | 64 | 915 | 7.8 | / | / | 16 | 2 | 100 |
